# Benefits of biomarker selection and clinico-pathological covariate inclusion in breast cancer prognostic models

**DOI:** 10.1186/bcr2633

**Published:** 2010-09-01

**Authors:** Fabio Parisi, Ana M González, Yasmine Nadler, Robert L Camp, David L Rimm, Harriet M Kluger, Yuval Kluger

**Affiliations:** 1 Department of Cell Biology, New York University Center for Health Informatics and Bioinformatics, New York University School of Medicine and Cancer Institute, 550 First Avenue, New York, NY 10016, USA; 2 Department of Pathology, Yale University School of Medicine, 333 Cedar Street, New Haven, CT 06520, USA; 3 Yale Cancer Center, Yale University School of Medicine, 333 Cedar Street, New Haven, CT 06520, USA; 4 Computer Science Department of the Universidad Autónoma of Madrid, Calle Francisco Tomás y Valiente, 11, Cantoblanco 28049, Madrid, Spain; 5 Department of Medicine, Yale University School of Medicine, 333 Cedar Street, New Haven, CT 06520, USA

## Abstract

**Introduction:**

Multi-marker molecular assays have impacted management of early stage breast cancer, facilitating adjuvant chemotherapy decisions. We generated prognostic models that incorporate protein-based molecular markers and clinico-pathological variables to improve survival prediction.

**Methods:**

We used a quantitative immunofluorescence method to study protein expression of 14 markers included in the Oncotype DX™ assay on a 638 breast cancer patient cohort with 15-year follow-up. We performed cross-validation analyses to assess performance of multivariate Cox models consisting of these markers and standard clinico-pathological covariates, using an average time-dependent Area Under the Receiver Operating Characteristic curves and compared it to nested Cox models obtained by robust backward selection procedures.

**Results:**

A prognostic index derived from of a multivariate Cox regression model incorporating molecular and clinico-pathological covariates (nodal status, tumor size, nuclear grade, and age) is superior to models based on molecular studies alone or clinico-pathological covariates alone. Performance of this composite model can be further improved using feature selection techniques to prune variables. When stratifying patients by Nottingham Prognostic Index (NPI), the most prognostic markers in high and low NPI groups differed. Similarly, for the node-negative, hormone receptor-positive sub-population, we derived a compact model with three clinico-pathological variables and two protein markers that was superior to the full model.

**Conclusions:**

Prognostic models that include both molecular and clinico-pathological covariates can be more accurate than models based on either set of features alone. Furthermore, feature selection can decrease the number of molecular variables needed to predict outcome, potentially resulting in less expensive assays.

## Introduction

Adjuvant systemic therapy for patients with breast cancer includes chemotherapy, anti-hormonal therapy and molecular targeted therapy. Selection of anti-hormonal and molecular targeted therapy is based on biological factors of individual tumors (presence/absence of hormone receptors and amplification/over-expression of human epidermal growth factor receptor (HER) 2). The decision whether to give chemotherapy and specifics of the chemotherapy regimens used are typically based on standard clinical and pathologic criteria (primarily tumor grade, tumor size, nodal involvement, patient age), in addition to receptor status. Given the variability in outcome in each risk category, much effort has been made to improve risk assessment strategies [1].

Assays that provide prognostic information to early stage breast cancer patients to eliminate unnecessary use of chemotherapy have been developed and validated. Among the breast cancer multi-marker predictors, two are fully commercialized; the RT-PCR-based Oncotype DX™ assay (Genomic Health, Redwood City, CA, USA) [2-6] and the 70-gene microarray based MammaPrint assay [7,8] (Agendia BV, Amsterdam, The Netherlands). The RT-PCR-based Oncotype DX™ assay is the most widely used in the USA. It has been validated in several studies, was recently endorsed by the American Society of Clinical Oncology (ASCO), and its cost is covered by third party payers, including Medicaid and Medicare. Samples are sent to a centralized location at Genomic Health for testing at a current cost of $3,460 per sample.

The Oncotype assay uses mRNA extracted from paraffin-embedded tumors to measure levels of 16 markers [9]. It has been validated in different cohorts [1]. Our purpose was to evaluate incorporation of standard clinico-pathological variables into models that include the Oncotype markers. To obtain a simplified protein-based assay, we employed a method of automated, quantitative analysis (AQUA) for these studies. This method has been used and validated in numerous prior breast cancer studies [10-12]. We derived models that were superior in outcome prediction to morphology alone or marker expression alone.

## Materials and methods

### Tissue microarray construction

Breast cancer tissue microarrays (TMAs) were constructed as previously described [13]. A cohort of 319 sequentially collected node-negative specimens and a separate cohort of 319 sequentially collected specimens from node-positive breast cancer patients from the Yale Department of Pathology Archives were cored. Specimens and clinical information were collected with Institutional Review Board approval.

By standard immunohistochemistry (IHC), estrogen receptor (ER) was positive in 52%, progesterone receptor (PR) in 46% and HER2 in 14% of specimens. Of those sampled, 26% were nuclear grade 3/3, 48% were nuclear grade 2/3, 18% were nuclear grade 1/3 and for 8% of the specimens nuclear grade score was missing. The mean tumor size was 2.9 cm and 59% were larger than 2 cm. A total of 72% were invasive ductal carcinoma, 14% were lobular carcinoma, and 14% had mixed or other histology. Specimens were resected between 1962 and 1983, and follow-up was between 4 months and 53 years (mean 12.6 years). Age at diagnosis was 24 to 88 years (mean 58 years).

Complete treatment history was not available for all patients. Most were treated with local irradiation. Node-negative patients were not given adjuvant systemic therapy. A minority of node-positive patients (about 15%) received chemotherapy, and about 5.6% received tamoxifen (ER-positive, post-menopausal, after 1978).

### Immunofluorescent staining

Staining was performed for AQUA analysis as previously described [10]. Primary antibodies are detailed in Table 1. All antibodies were carefully validated, as described previously [14-16]. Goat anti-mouse (or anti-rabbit) horseradish peroxidase-decorated polymer backbone (Envision; Dako, Carpinteria, CA, USA) was used as a secondary reagent, and Cy5-tyramide (Perkin Elmer Life Science, Waltham, MA, USA) was used to visualize the target. Anti-cytokeratin antibodies conjugated to Alexa-488 were used to create a tumor mask, to distinguish malignant cells from stroma. Nuclei were visualized using 4',6-diamidino-2-phenylindole. Staining of representative histospots for ER, PR and HER2 have been published elsewhere [17]. Staining for ER and PR was uniformly nuclear, and staining for HER2 was uniformly membranous, as seen with routine IHC.

**Table 1:** Primary antibodies and the company that supplied them

Protein (species)	Company
BCL2 (mouse)	Dako, Carpinteria, CA, USA
BAG1 (mouse)	Chemicon, Millipore, Billerica, MA, USA
BIRC5 (rabbit)	Novos Biological, Littleton, CO, USA
MKI67 (mouse)	BD pharmingen, San Jose, CA, USA
CD68 (Cd68)	GeneTex, Irvine, CA, USA
MYBL2 (rabbit)	GeneTex, Irvine, CA, USA
MMP11 (mouse)	Chemicon, Millipore, Billerica, MA, USA
GRB7 (GRB7 rabbit)	Santa Cruz, Santa Cruz, CA, USA
AURKA (rabbit)	Cell signaling,Danvers, MA, USA
GSTM1 (mouse)	Novus Biologicals, Littleton, CO, USA
CCNB1 (mouse)	Novus Biologicals, Littleton, CO, USA
CTSL2 (mouse)	R&D, Minneapolis, MN, USA
ESR1 (mouse)	Dako, Carpinteria, CA, USA
PGR (mouse)	Dako, Carpinteria, CA, USA
ERBB2 (rabbit)	Dako, Carpinteria, CA, USA

### Automated image acquisition and analysis

Images were acquired for AQUA, as extensively described previously [13]. Briefly, multiple monochromatic, high-resolution (1,024 × 1,024 pixels, 0.5 μm) grayscale images were obtained for each histospot, using the 10× objective of an Olympus AX-51 epifluorescence microscope (Olympus, Center Valley, PA, USA) with an automated microscope stage and digital image-acquisition driven by custom program and macro-based interfaces with IPLabs software (Scanalytics Inc., Fairfax, VA, USA). Images were analyzed using algorithms that have been previously described [10]. Data were expressed as the average signal intensity per unit area of tumor mask on a scale of 0 to 255.

### Statistical analysis

We measured protein levels of 14 of the 16 oncotype markers. Strong correlations were found between ER, PR and HER2 scores generated by pathologist IHC-based scoring. The significance for the Spearman correlations for ER, PR and HER2 are *P *< 10^-90 ^for all three variables, (*P *= 0.7264, 0.6353 and 0.7148, respectively). As pathologic values for ER, PR and HER2 by IHC are typically readily available at the time of initial diagnosis of breast cancer at no additional cost, the results described in this paper use the pathologist scores only. We constructed multivariate Cox proportional hazards models to analyze this set of protein markers in addition to standard clinical markers, which included ER, PR and HER2 obtained using standard IHC. We derived models predictive of 15-year breast cancer-specific survival. We performed these analyses for three different marker sets: the set of 14 markers, where the levels of ER, PR and HER2 were obtained using standard IHC and other markers by AQUA; the combination of these 14 protein markers (11 measured by AQUA and ER, PR and HER2 by IHC) with the remaining four clinico-pathological variables - nodal status, tumor size, nuclear grade, and age; and the seven clinico-pathological variables - nodal status, tumor size, nuclear grade, age, ER, PR and HER2. We binarized patient age at 50 years.

The AQUA scores and IHC variables were not normally distributed, as expected. For example, HER2 IHC scores were predominantly negative, and the AQUA scores for the HER2 adaptor protein GRB7 were predominantly low. This is consistent with what is known about the biology of these markers, and we therefore used the raw average of scores for all markers.

### Incorporation of the Nottingham Prognostic Index

The number of cases (with no missing AQUA values) in the standard, clinically-used low, intermediate and high Nottingham Prognostic Index (NPI) groups of our cohort is 124, 265, and 120, respectively. To increase sample size we split the cohort binarizing patients by an NPI of 4.4. We performed Cox proportional hazards analyses on these two subpopulations using the 18 protein and clinico-pathological variables.

### Nested cross-validation for model selection and model assessment

To accomplish model size reduction via feature selection and assess performance of models in an unbiased fashion, we employed a nested cross-validation procedure [18-20]. Specifically, we performed 100 times 10-fold cross-validation for model validation, using a nested 10-fold cross-validation procedure for feature and model selection. A pseudocode is provided in Table 2.

**Table 2:** Pseudocode of nested cross-validation for model selection and model assessment

Repeat 100 times:				

	Divide the data into 10 outer folds			
	Repeat 10 times:			

		Keep 1 outer fold for testing		
		Select the remaining 9 outer folds for training		
		Divide the 9 outer training folds into 10 inner folds		
		Repeat 10 times:		

			Keep 1 inner fold for testing	
			Select the remaining 9 inner folds for training	
			Move all variables into the list of available variables	
			Create an empty list of nested model variables	
			Iterate this backward selection procedure until only 1 variable is left in the list of available variables:	

				Train Cox models on the inner training set. Each Cox model contains all available variables except of 1 variable at a time
				Select the variable that contributes the least to the model likelihood
				Move the selected variable from the list of available variables to the top of the list of nested model variables

			Move the last available variable to the top of the list of nested model variables	
			Iterate over the list of nested variables:	

				Train the Cox model containing the present variable and the variables above it in the list of nested variables using the inner training set.
				Evaluate the average time-dependent area under the receiver operating characteristic curve (ATD-AUCROC) *h *of the present Cox model using the 1 inner testing fold.
				Record the variable usage U in the present Cox model and the size *n *of the model. U_X_(v_m_) = 1 if v_m _is in model X, 0 otherwise.

		Estimate:		
		- the expected model size <n> = Σ_X_(h_X _n_X_)/Σ_X_(h_X_)		
		- the (inner) variable stability score for each variable v_m_: <v_m_> = Σ_X_(h_x _U_X_(v_m_))/Σ_X_(h_x_)		

	Train the Cox model containing the most stable <n> variables using the outer training set.			
	Evaluate the ATD-AUCROC *k *of the present Cox model using the 1 outer testing fold.			
	Record the variable usage T in the present Cox model and the size *s *of the model.			
	T_X_(v_m_) = 1 if v_m _is in model X, 0 otherwise.			

### Partitioning of data for nested cross-validation

To prevent overfitting, we effectively partitioned the data into three: a feature extraction training subset (inner training set); a model size selection and variable stability evaluation subset (inner testing set); and an outer test set for performance estimation of models trained on the outer training set, which comprised the inner training and testing sets. Specifically, we partitioned the data into 10 non-overlapping, balanced subsets of cases (outer folds). Following a standard n-fold cross-validation approach, each fold was used once as the outer testing set where the remaining folds were used as the outer training set. Similarly, at each iteration of the outer 10-fold cross-validation we partitioned the data of the outer training folds (90% of the overall data) into 10 folds to be used in an inner 10-fold cross validation loop.

### Variable and model size selection

We used the inner training set to train reduced nested Cox models with decreasing numbers of variables from n-1 variables to one variable. These nested models were determined by a backward feature elimination procedure that iteratively removed variables with the smallest contribution to the model likelihood. We then used the inner test fold to compute the inner performance score for each of the nested models trained on the inner training folds and selected the reduced model with the highest score. The performance score of these Cox models was evaluated using the area under the staircase receiver operator characteristic curves (AUCROC) at the time of each event in the testing set and we averaged these AUCROCs across all these events. We refer to this measure as the average time-dependent AUCROCs across all death events. The average time-dependent AUCROC measure is a variant of the weighted average of time-dependent ROC curve approaches [21-23]. We compute the AUCROC from the staircase ROC curve to avoid overestimation associated with convex hull or trapezoidal interpolation procedures.

Each iteration of the inner 10-fold cross-validation returns one reduced model. We used these 10 reduced models to compute the expected model size. The expected model size is the weighted average of the size of these models using as weights their respective inner average time-dependent AUCROCs. Similarly, the stability of each variable was determined as the fraction of reduced models containing the variable; to take performance into account we defined an alternative variable stability score by summing the average time-dependent AUCROCs of the reduced models that include the variable.

### Model selection and validation

**Figure 1:**
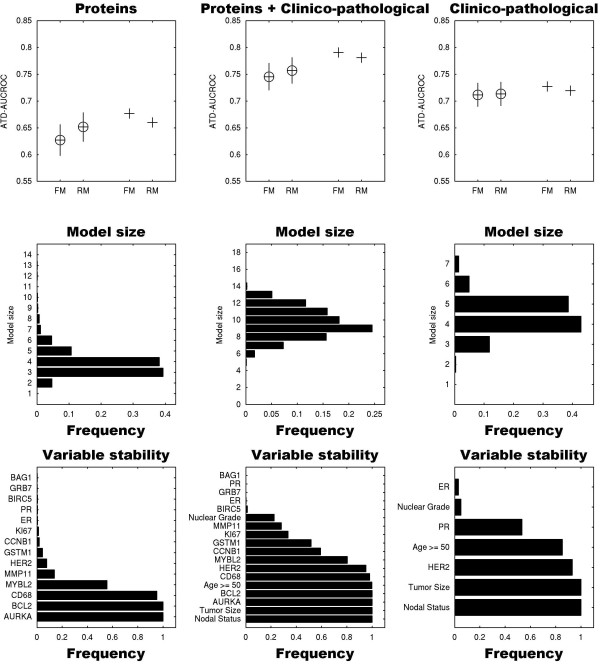
**Performance, model size distribution and variable stability of reduced models for predicting 15-year breast cancer-specific survival**. Upper row: The average time-dependent area under the receiver operator characteristic curve (ATD-AUCROC) performances of the full Cox models (FM) and reduced models (RM) derived utilizing 14 of the proteins included in the Oncotype DX assay (left column), the 18-variable full model that incorporates these 14 markers with four additional clinico-pathological variables (middle column) and seven standard clinico-pathological variables (right column) are denoted by circles. The corresponding performances on the training sets are denoted by plus signs. Error bars span ± 1 standard deviation from the average performance of the models. Combining protein plus clinico-pathological variables improved model performance, and variable reduction shown in the reduced models resulted in further improvement. Middle row: The sizes of the 15-year survival reduced Cox models were derived from the expected model size distributions. Bottom row: The variables incorporated in these reduced models were chosen according to their stability (frequency) in the nested cross-validation procedure. Distribution of model sizes and frequency-based stability were derived from the reduced models trained on the outer training set. For example, the average size distribution of the reduced models derived from the protein only variables (left column) is four, and thus the final reduced model includes AURKA, BCL2, CD68 and MYBL2. ER, estrogen receptor; HER, human epidermal growth factor receptor; PR, progesterone receptor.

For each iteration of the outer 10-fold cross-validation we used the outer training set to train: a reduced Cox model consisting of the expected model size number of variables with the largest variable stability score; and a full Cox model comprising all variables. Finally, the full and reduced models are assessed both on the outer training and testing sets (training and testing performances are shown in red and black respectively in Figure 1).

### Statistical comparison of models

To compare the distributions of Cox model average time-dependent AUCROCs of the 100 times 10-fold cross-validation (e.g. full or reduced models) we applied two-sided Mann-Whitney U-tests.

## Results

### Risk of death models that incorporate both protein markers and clinical/pathological variables

We employed a method of quantitative immunofluorescence to derive multivariate Cox proportional hazards models for 15-year survival. We had a total of 18 variables; 14 protein markers from the Oncotype panel (SCUBE2 and CTSL2 were omitted due to a lack of commercially available, technically reproducible antibodies for immunofluorescent staining of paraffin-embedded specimens) and four clinico-pathological markers. ER, PR and HER2 were assessed by IHC. Many of these variables were univariately prognostic as shown in Table 3. We assessed the prognostic ability of this model and other models presented below by average time-dependent AUCROC. The mean cross-validated average time-dependent AUCROC of this 18-covariate model is 0.746 at 15 years. We compared this 18-covariate model with a 14-covariate Cox proportional hazards model consisting of the same protein markers (including ER, PR and HER2), but excluding nodal status, size, nuclear grade and patient age. The average time-dependent AUCROC of this protein-based model is 0.627. The mean of the distribution of average time-dependent AUCROCs of the 18-covariate models obtained by 100 10-fold cross-validations is significantly higher than the corresponding distribution mean of the 14-covariate protein-based model (*P *< 10^-10^). The performances of the full models are shown in Figure 1, upper row (FMs). We then derived a Cox proportional hazards model with the seven standard clinico-pathological variables only (ER, PR and HER2 by IHC, plus nodal status, size, grade and age). The mean average time-dependent AUCROC of the seven-covariate clinico-pathological model is 0.712. This is significantly lower than the mean of the composite 18-covariate model (*P *< 10^-10^) and significantly higher than the mean of the 14-covariate protein model (*P *< 10^-10^).

**Table 3:** Univariate analysis for each of the 18 markers included in the full model of Figure 1

Variable	95% CI		*P *value
**[Pathology]**			
Tumor size	1.064	1.200	0.0003
Age <50 years	0.959	2.075	0.0719
Nuclear grade	1.017	1.676	0.0353
Nodal status	1.717	3.456	2.3 × 10^-7^
ER (IHC)	0.709	0.926	0.0017
PR (IHC)	0.751	0.986	0.0281
HER2 (IHC)	1.018	1.379	0.0337
**[AQUA]**			
AURKA	1.008	1.019	0.0001
BAG1	0.986	1.008	0.5826
BCL2	0.988	0.999	0.0084
BIRC5	0.999	1.023	0.0642
CCNB1	0.968	1.060	0.5854
CD68	0.975	1.017	0.6866
GRB7	1.006	1.019	0.0008
GSTM1	0.996	1.030	0.1412
KI67	0.994	1.041	0.1722
MMP11	0.996	1.018	0.2349
MYBL2	0.984	1.016	0.9950

For each marker a univariate Cox proportional hazards model is fit to the data using the entire cohort. The 95% confidence interval (CI) is shown together with the log-likelihood test *P *value. Estrogen receptor (ER), progesterone receptor (PR) and human epidermal growth factor receptor (HER) 2 were measured by immunohistochemistry (IHC).

### Variable reduction

We sought to simplify the three models (proteins with clinico-pathological and either group alone). We employed stability-based backward feature selection (described above) to derive compact Cox proportional hazards models nested within each of these three models. The cross-validated performances of the 18-variable (protein and clinico-pathological) full models and reduced models selected from these 18-variables were assessed by the average time-dependent AUCROC measure, denoted by circles (upper row, Figure 1). The corresponding performances of the training sets are denoted by plus signs.

Application of the robust backward feature selection described above eliminated on average eight of the least robust features. The reduced models selected were assessed employing the average time-dependent AUCROC score to the external validation sets. The mean average time-dependent AUCROC distribution of these reduced models is 0.757, significantly higher than the corresponding distribution of the 18-variable full models (*P *= 0.0021606, Mann-Whitney U-test). The nested cross-validated procedure culminates in a final model that excludes BAG1, PR, GRB7, ER, BIRC5, nuclear grade, MMP11 and KI67. The variables retained in this model include the 10 variables with highest stability score (bottom row, middle column, Figure 1). Furthermore, the average time-dependent AUCROC score distribution of reduced models derived from the 18 clinico-pathological and protein variables, is significantly higher than the corresponding distribution of reduced models derived from clinico-pathological variables alone (*P *< 10^-10^).

We similarly analyzed the same 14 proteins, but excluded the clinico-pathological variables (nodal status, tumor size, age, nuclear grade). The average size of the 15-year survival Cox proportional hazards model (middle row, left column, Figure 1) indicates that for an assay based on these protein covariates, it is optimal to keep only the four most robust variables, AURKA, BCL2, CD68 and MYBL2, in a simplified survival model. The average time-dependent AUCROC of these reduced models is 0.651, significantly higher than the corresponding average of the full models (*P *= 7.32 × 10^-9^). The reduced protein-based models, although superior to the full protein-models, significantly underperform with respect to the combined protein and clinico-pathological models. These protein-only models also underperform relative to models based on standard clinico-pathological variables alone (average time-dependent AUCROC = 0.711) and its nested reduced models (average time-dependent AUCROC = 0.713), right column, Figure 1.

### Prognosis for low and high NPI populations

**Figure 2:**
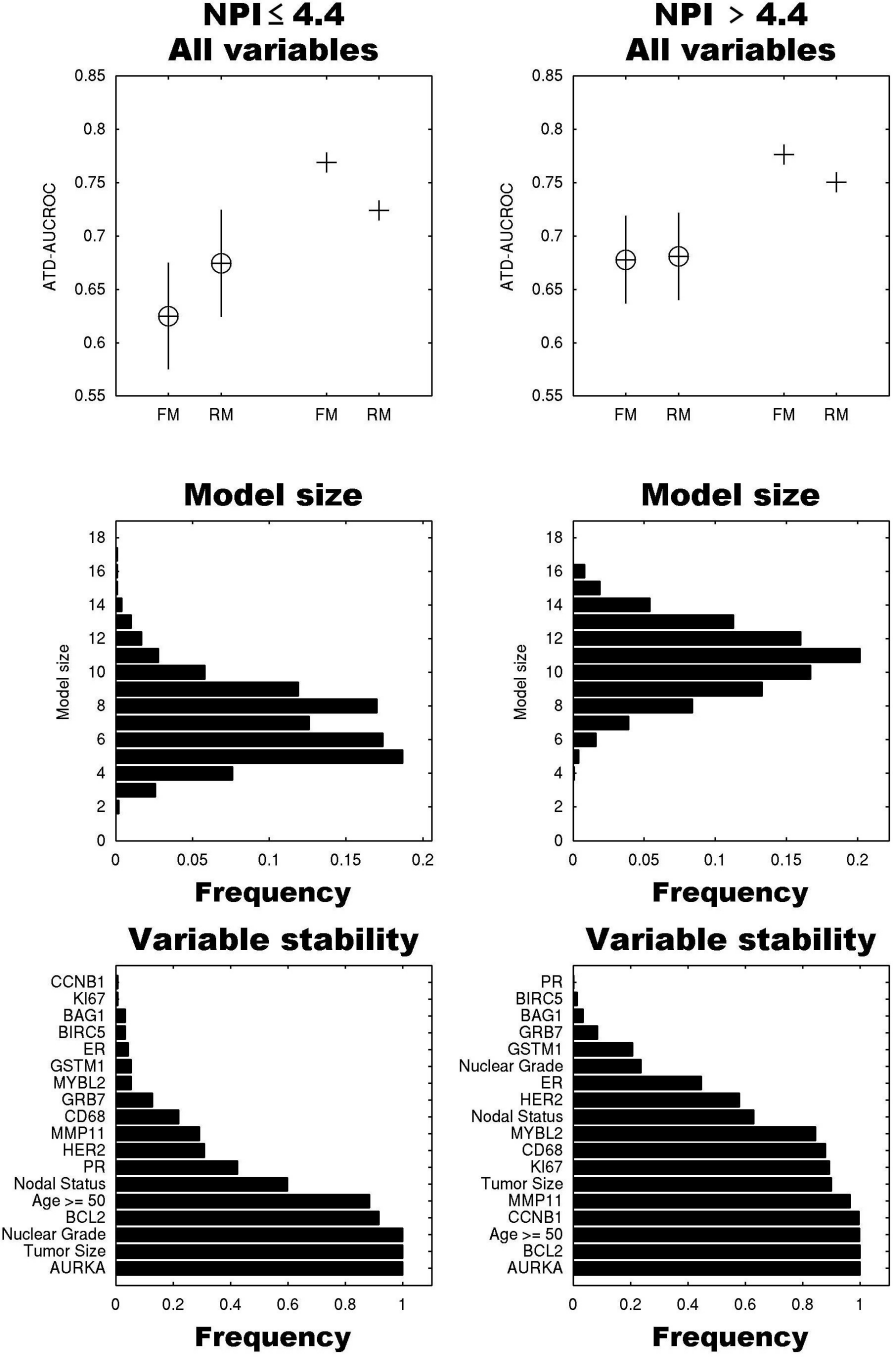
**Performance, model size distribution and variable stability of reduced models as described in Figure 1 for lower NPI and higher NPI risk groups**. Left column: Patients with an Nottingham Prognostic Index (NPI) of more than 4.4. Right column: NPI of 4.4 or less. The final reduced model (RM) for the lower NPI group consists of 7 variables, whereas the final reduced model for the higher NPI group consists of 11 partially overlapping variables. For example, CCNB1 is one of the most robust variables in the higher NPI group, but is the least robust variable in the lower NPI group. ATD-AUCROC, average time-dependent area under the receiver operator characteristic curve; ER, estrogen receptor; FM, full models; HER, human epidermal growth factor receptor; PR, progesterone receptor.

We next sought to determine whether in each NPI category the panel of 18 markers can be reduced and whether sets of survival predictors for different NPI subpopulations vary. The number of cases with no missing values in the low, intermediate and high NPI groups was 124, 265, and 120, respectively. Due to the small sample size of the low and high NPI groups, we did not use the standard NPI cut-points of 3.4 and 5.4, but binarized the population at the midpoint of this NPI range (4.4). We applied robust backward elimination for lower and higher NPI groups (Figures 2a and 2b, respectively). Simplification of the models results in higher assessment scores in both groups and an expected model size of 7 and 11 variables, respectively. The difference between the means of the average time-dependent AUCROC distributions of full models (0.625) and reduced models (0.675) in the lower NPI group reached significance (*P *< 10^-10^). These distributions are indistinguishable in the higher NPI group (*P *= 0.49). Some markers such as CCNB1, KI67 and MYBL2 are not included in the lower NPI reduced model but are present in the reduced model of the higher NPI group. This indicates that one could tailor simpler/cheaper multi-protein predictors to populations stratified by clinico-pathological variables.

### Prognosis for node-negative, hormone receptor-positive population

**Figure 3:**
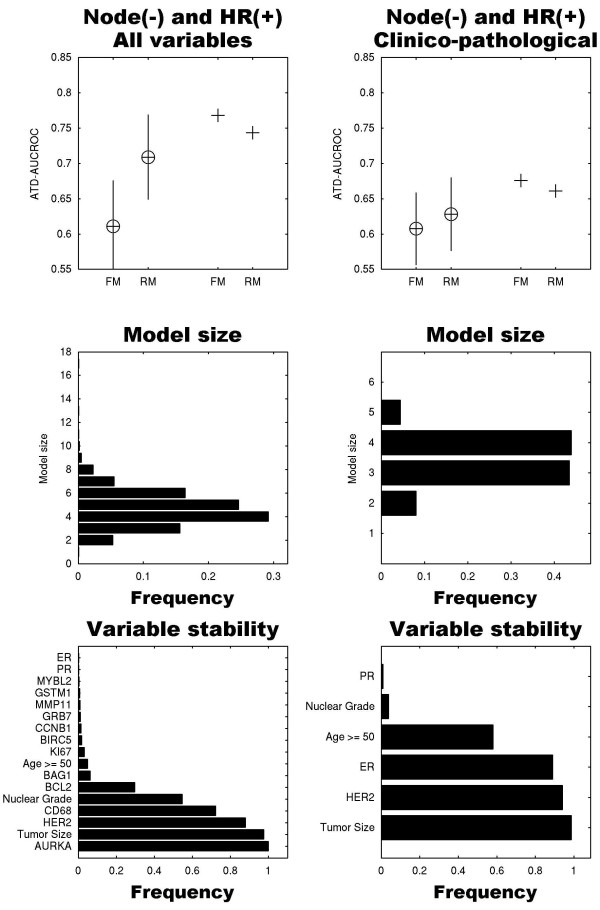
**Performance, model size distribution and variable stability of reduced models as described in Figure 1 for node-negative (node(-)) and hormone receptor positive (+) subpopulation**. We included patients whose tumors were estrogen receptor (ER) positive, progesterone receptor (PR) positive or both. The left column shows the models for all variables excluding nodal status, and the right column shows models for the clinico-pathological variables alone (tumor size, nuclear grade, age, human epidermal growth factor receptor (HER) 2, ER, PR). The compact, reduced model (RM) derived from molecular and clinico-pathological covariates dramatically outperformed the full models (FM), and included AURKA, tumor size, HER2, CD68 and nuclear grade. ATD-AUCROC, average time-dependent area under the receiver operator characteristic curve.

We questioned whether we can compress the full 18-variable model for the subpopulation of node(-), hormone receptor (+) breast cancers, as many of these patients in the USA are tested using the Oncotype DX™ screen. The full model in this case consists of 17 variables, because the nodal status variable is fixed, but ER and PR variables are either positive or negative, as long as at least one of the two hormone receptors is positive. Applying robust backward selection in a nested cross-validated fashion resulted in a highly compact model consisting of five variables: AURKA, tumor size, HER2, CD68 and nuclear grade (Figure 3). The mean of the average time-dependent AUCROC distribution (0.71) is significantly higher than the mean of the full 17-variable model (0.63, *P *< 10^-10^). The full and reduced models of the clinico-pathological variables alone applied to this sub-population held inferior performances (Figure 3, right column).

## Discussion

We measured protein expression levels of 14 of the 16 oncotype markers in primary tumors from 638 breast cancer patients with 15-year follow-up, using AQUA. This method has now been well established and is used by many laboratories [24-32]. Measurements can be conducted on whole specimens or TMAs. Many of the oncotype markers were independently prognostic [14-16]. We assessed the added value of each oncotype marker in combination with standard clinical and pathological variables, including ER, PR and HER2 evaluated by eye using routine IHC. Our studies indicate that a multivariable survival model including both molecular markers and standard clinical/pathological markers is significantly superior to a model based on either group of variables alone. Moreover, with judicious subset selection of the combined set of clinico-pathologic variables and oncotype markers, we derived a more compact test with better cross-validated prognostic value. We also showed that when splitting the patient cohort into two groups of NPI of 4.4 or less and more than 4.4, we obtain different marker subsets in these groups. Finally, we showed that for the node-negative, hormone receptor-positive subpopulation, a compact model consisting of only three proteins of the panel of 14 (AURKA, HER2, CD68), tumor size and nuclear grade is superior to a full model consisting of these 14 variables with the additional standard clinico-pathological variables.

Optimal staging of breast cancer patients is primarily necessary for identifying individuals in need of adjuvant chemotherapy. The seven clinico-pathological variables included in our model are typically readily available on all patients, and can be incorporated into molecular assays at no additional cost. The performance of our reduced nested models converges at a value close to 0.757 if we include both molecular and clinico-pathological covariates and drops to 0.651 if we exclude the clinico-pathological variables. Oncotype assays by RT-PCR of the 16 molecular variables in other patient cohorts are reportedly associated with AUCROCs in the same range. For example, using the oncotype RS, Goldstein et al. found that for recurrence at five-years, ROC analysis results in an AUCROC of 0.69 [33]. Direct comparisons between oncotype results and our findings are not possible given the differences in patient cohorts, treatment patterns, available clinical endpoints and differences in model evaluation methods. For example, the oncotype assay was developed for a hormone receptor-positive population treated with tamoxifen and progression-free survival was the primary endpoint. The primary endpoint in our studies was overall survival and the cohort included hormone receptor positive and negative patients. Our purpose was not to conduct a head to head comparison of our method to the oncotype method, and it is unclear how the protein-based AQUA scores relate to the RT-PCR measures of mRNA obtained by oncotype. However, our work further validates the use of oncotype markers by confirming their prognostic value by studying them at the protein level using different technology. A limitation of this study is that we were unable to obtain a cohort in which the Oncotype Dx test was performed to facilitate head to head comparison, and further validation of our protein-based models in an independent cohort is warranted.

The performance of our reduced models suggests that we can considerably simplify our original models of 18 variables. The expected model size with the highest performance level consists of 10 of the most robust predictive variables and is comprised of four clinico-pathological variables and six additional proteins: nodal status, tumor size, AURKA, BCL2, age, CD68, HER2, MYBL2, CCNB1 and GSTM1. Thus, use of a smaller subset of variables can further decrease the cost of molecular testing.

Further extension of this approach by sub-setting the cohort into low- and high-risk groups using an NPI score of 4.4, which is readily available after standard surgery at no additional cost, revealed that the marker subset with optimal performance in the lower NPI group was different than the subset in the higher NPI group. The seven variables in the reduced model for the lower NPI overlap only in part with the 11 variables in the reduced model of the higher NPI group. The reduced model of the node(-) and hormone receptor (+) subpopulation consists of only five variables, of which three are proteins (AURKA, HER2, CD68).

## Conclusions

In our cohort, addition of clinico-pathological variables to the proteins associated with quantitative RT-PCR Oncotype test added significant prognostic value to the proteins alone. A compact model based on a subset of these proteins and clinical variables is superior to the entire model. Marker subsets with the highest prognostic ability in high- and low-risk NPI categories are not identical, therefore personalization of this type of assay based on readily available clinico-pathological variables can result in cost reduction without compromising accuracy.

## Abbreviations

AQUA: automated quantitative analysis; AUCROC: area under the receiver operator characteristic curve; ER: estrogen receptor; HER: human epidermal growth factor receptor; IHC: Immunohistochemistry; NPI: Nottingham Prognostic Index; PR: progesterone receptor; RT-PCR: reverse transcription polymerase chain reaction; TMA: tissue microarray.

## Competing interests

Drs. Camp and Rimm are founders, stockholders, and consultants to HistoRx, a private corporation to which Yale University has given exclusive rights to produce and distribute software and technologies embedded in AQUA; Yale University retains patent rights for the AQUA technology. Dr. Rimm is also a stockholder and consultant to Metamark Genetics Inc.

## Authors' contributions

YK and HK initiated the project. FP, AG and YK performed all the computational aspects of the projects. YK, HK and FP drafted the manuscript. YN performed the AQUA experiments and preprocessed the data. RLC and DLR developed the AQUA technology and provided the analysis for HER2, ER and PR. All authors read and approved the manuscript.
